# Sculling Compensation Algorithm for SINS Based on Two-Time Scale Perturbation Model of Inertial Measurements

**DOI:** 10.3390/s18010282

**Published:** 2018-01-18

**Authors:** Lingling Wang, Li Fu, Ming Xin

**Affiliations:** 1 School of Automation Science and Electrical Engineering, Beihang University, Beijing 100191, China; wangling0908@buaa.edu.cn; 2 Department of Mechanical and Aerospace Engineering, University of Missouri, Columbia, MO 65211, USA; xin@missouri.edu

**Keywords:** sculling error, singular perturbation, two-time scale perturbation model, velocity update, pseudo sculling, inertial measurement

## Abstract

In order to decrease the velocity sculling error under vibration environments, a new sculling error compensation algorithm for strapdown inertial navigation system (SINS) using angular rate and specific force measurements as inputs is proposed in this paper. First, the sculling error formula in incremental velocity update is analytically derived in terms of the angular rate and specific force. Next, two-time scale perturbation models of the angular rate and specific force are constructed. The new sculling correction term is derived and a gravitational search optimization method is used to determine the parameters in the two-time scale perturbation models. Finally, the performance of the proposed algorithm is evaluated in a stochastic real sculling environment, which is different from the conventional algorithms simulated in a pure sculling circumstance. A series of test results demonstrate that the new sculling compensation algorithm can achieve balanced real/pseudo sculling correction performance during velocity update with the advantage of less computation load compared with conventional algorithms.

## 1. Introduction

There are two key calculations in a typical strapdown inertial navigation system (SINS), namely, the attitude update and velocity update algorithms. The total acceleration in SINS is expressed in terms of the specific force, gravity and centrifugal acceleration. The velocity update algorithm of SINS is a process of integrating total acceleration in the navigation frame. During the velocity update process, the velocity differential equation can be obtained by transforming the specific force from the body frame to the navigation frame based on the attitude update result, and including other accelerations.

For the attitude update, many algorithms with high accuracy have been developed [1,2,3]. For the velocity update, many research works focus on multi-sensor information fusion based on various filtering techniques to achieve high accuracy velocity for alignment and navigation of SINS. In order to improve velocity estimation for alignment, an adaptive unscented Kalman filter with the aid of a star sensor is proposed in [4]. A 12-state Kalman filter is used to estimate the velocity error for the stationary self-alignment in [5]. In [6], a method using the Kalman filter based on integrating gravitational apparent motion to form apparent velocity is designed to reduce the random noise of the observation vectors for self-alignment. A tightly coupled SINS/GPS with the extended Kalman filter is utilized in [7] to improve the attitude and velocity update accuracy with the observability analysis of the whole system. Although the above methods achieve better performances, they all need other observable information with higher precision as measurement vectors. Recently, a dual Kalman filter has been developed in [8,9] to estimate the unknown input and states by using sparse noisy acceleration measurements. The outcome indicates that this method can achieve optimal performance in the presence of colored measurement and modelling errors, and it is effective for the state estimation of the metallic structures under low frequency vibrations due to unknown input forces. However, the aircraft considered in this paper works in a more complex environment than the metallic structure in that it undergoes not only low frequency vibrations but also wideband vibrations.

In a dynamic oscillating environment, the conventional velocity update usually generates the delta velocity quantity and causes the navigation system to degrade if there is no sculling compensation. Another method to enhance the velocity accuracy is to improve the numerical calculation of the velocity differential equation. In the past few decades, considerable researches have been focused on developing highly accurate and highly efficient velocity update algorithms. Savage [10,11] proposed a two-speed velocity update algorithm and a velocity translation vector. Sculling error compensation was computed with high speed by integrating the exact velocity translation vector differential equation, while the velocity was updated in a moderate-speed process. To further improve the accuracy of the two-speed velocity update algorithm, Wu et al. [12] developed a simpler dual quaternion (DQ) algorithm to determine the rotation and apparent velocity as a whole. Chelnokov [13] proved that the DQ solutions were stable in Lyapunov’s sense and the two-speed DQ algorithm achieved the third and fourth order of accuracy. In [14,15], geometry algebra and Lie group methods were used to construct the two-speed velocity update algorithms with comparable accuracy to the DQ algorithm. In addition, a simplified parallel velocity error compensation algorithm was proposed in [16,17] based on the two-speed velocity update concept and executed on a single-chip field programmable gate array (FPGA).

For the above velocity update algorithms, the sculling motion was assumed to oscillate with constant amplitude and frequency, and the signals from the accelerometers and gyroscopes were measured with white noise. However, in real dynamic environments, there exist random sculling motion and nonwhite stochastic noise in inertial sensor measurements. Previous works cannot mitigate the effect of these errors, thus limiting the velocity update accuracy in real applications. In order to overcome this limitation, Wang et al. [18] presented a nonlinear robust multiple integrator algorithm to estimate velocity from the measurements of accelerometers based on finite-time stability and singular perturbation technique. Furthermore, with the assumption that the derivative of acceleration satisfies a particular perturbation model, the presented algorithm proved that the velocity estimation was not only robust to the nonwhite noise but also finite-time stable. However, the particular perturbation model makes this method difficult to extend to the velocity update used in the SINS algorithm design. In [19], a new coning correction algorithm, based on the two-time scale perturbation model of the angular rate, was proposed for the attitude update algorithm with non-ideal angular rate information. It is proved valid for improving accuracy with reduced computations of the attitude update in SINS undergoing stochastic coning environments.

On the other hand, equivalency between the strapdown coning and the sculling correction algorithm has been proved in [20]. Additionally, the singular perturbation model can be used to analyze the vehicle dynamics equipped with an aerodynamic vectoring feature and has been applied in designing aerospace guidance and control systems [21,22]. In this paper, we extend the previous work to design the new velocity update algorithm based on singular perturbation.

In order to decrease the dynamic integration error of velocity update under vibration and other complicated environments, a new sculling compensation algorithm for the velocity integration based on a two-time scale perturbation model of inertial information is presented in this paper. An analytical expression for the sculling error from the specific force integration process is derived in Section 2. This expression can provide a solution to the dynamic sculling velocity error compensation. Then, we analyze the frequency spectrum of the specific force under a real vibration environment to provide a basis for the two-time scale perturbation model. Based on the analytical expression and two-time scale perturbation model of the specific force, a new sculling error compensation algorithm is derived in Section 3 combined with our previous work on attitude update [19]. A gravitational search optimization method is used to determine the perturbation parameters in the sculling error correction term. The performance of the new sculling compensation algorithm is evaluated and compared with the conventional sculling compensation algorithm under stochastic sculling motions in Section 4. The simulation results indicate that the proposed sculling compensation algorithm improves the velocity update accuracy and decreases computation load as well.

## 2. Sculling Error Formula in Velocity Update

The following relevant coordinate frames about navigation were used:

- The body coordinate frame (*b*-frame) is the strapdown inertial sensor frame. It is a right-handed frame with the origin at the center of vehicle gravity. Its X-axis, Y-axis and Z-axis are aligned with the roll, pitch and yaw directions of the vehicle, respectively.
- The navigation coordinate frame (*n*-frame) is a local geographic frame with its X-axis, Y-axis, and Z-axis aligned with the directions of north, east, and the local vertical (down), respectively.
- The earth coordinate frame (*e*-frame) has an Earth-fixed angular geometry relative to the earth with the origin at the center of earth. The X-axis passes through the Greenwich meridian and the Y-axis is perpendicular to X-axis in the equatorial plane. Z-axis points toward the north pole.
- The inertial coordinate frame (*i*-frame) is a non-rotating frame with its origin at the center of earth. Its X-axis is in the equatorial plane pointing towards the vernal equinox and the Z-axis points toward the north pole. The Y-axis completes the right-handed frame.

According to the mechanization process of SINS, the velocity update equation in *n*-frame is known as:

(1) $${\dot{v}}^{n}=C_{b}^{n}f^{b}-(2\omega_{ie}^{n}+\omega_{en}^{n})\times v^{n}+g^{n}$$

where $C_{b}^{n}$ is a direction cosine matrix that transforms a vector from *b*-frame to *n*-frame. $f^{b}$ is the specific force vector measured by the strapdown accelerometer in *b*-frame. $\omega_{ie}^{n}$ denotes the earth angular rate relative to *i*-frame expressed in *n*-frame, and $\omega_{en}^{n}$ is the angular rate of *n*-frame with respect to *e*-frame expressed in *n*-frame. $v^{n}$ is the vehicle velocity in *n*-frame and ${\dot{v}}^{n}$ is the velocity change over the velocity update interval. $g^{n}$ is the gravity vector expressed in *n*-frame.

It can be seen that the velocity update Equation (1) contains three terms: the transformed specific force term, the Coriolis acceleration, and the apparent gravity, respectively. The second and third terms vary slowly and smoothly during one navigation period and can be calculated using the linear interpolation method over a small time period [23]. However, under the condition of large angular motions, the attitude changes rapidly in the update interval, which can cause large errors in the specific force integration and reduce the velocity update accuracy. Therefore, the velocity update algorithm must account for the error caused by frame rotation to meet the requirement of precise navigation [24,25]. Let $f^{n}$ be the specific force vector with respect to *n*-frame and utilize the strapdown accelerometer output $f^{b}$, then

(2) $$f^{n}=C_{b}^{n}f^{b}$$

Integrate the specific force in *n*-frame over each sculling update interval from $t_{k}$ to $t_{k+1}$ as follows

(3) $$u^{n}=\int_{t_{k}}^{t_{k+1}}C_{b}^{n}f^{b}dt$$

where $t_{k}$ and $t_{k+1}$ represent the beginning time and ending time of one sculling update interval. $u^{n}$ denotes velocity increment in *n*-frame from $t_{k}$ to $t_{k+1}$.

Define $C_{n(k)}^{n(k+1)}$ as the direction cosine matrix from *n*-frame at $t_{k}$ to *n*-frame at $t_{k+1}$. The direction cosine matrix from *b*-frame at $t_{k}$ to *n*-frame at $t_{k}$ is denoted by $C_{b(k)}^{n(k)}$. Denote $C_{b(t)}^{b(k)}$ as the direction cosine matrix from *b*-frame at t to *b*-frame at $t_{k}$. Then, the direction cosine matrix from *b*-frame at t to *n*-frame at $t_{k+1}$ becomes

(4) $$C_{b}^{n}=C_{n(k)}^{n(k+1)}C_{b(k)}^{n(k)}C_{b(t)}^{b(k)}$$

Letting $C=C_{n(k)}^{n(k+1)}C_{b(k)}^{n(k)}$ and substituting Equation (4) into Equation (3) yields

(5) $$u^{n}=C\int_{t_{k}}^{t_{k+1}}C_{b(t)}^{b(k)}f^{b}dt$$

where $C_{b(t)}^{b(k)}=I+[\phi \times ]+0.5{\left[\phi \times \right]}^{2}+\cdots$, ϕ denotes the rotation vector defining the body attitude from $t_{k}$ to *t* [4,17]. ϕ× is the skew symmetric matrix of the rotation vector ϕ. Therefore, Equation (5) can be derived as

(6) $$u^{n}=C\int_{t_{k}}^{t_{k+1}}\left(I+[\phi \times ]+0.5{\left[\phi \times \right]}^{2}+\cdots \right)f^{b}dt$$

For most SINS mechanization, the second cross-product term and other higher order terms can be assumed small enough to be negligible. Thus, $u^{n}$ can be approximated by

(7) $$u^{n}=C\left(\int_{t_{k}}^{t_{k+1}}f^{b}dt+\int_{t_{k}}^{t_{k+1}}\phi \times f^{b}dt\right)$$

Over a small time interval, the rotation vector ϕ can be approximated by

(8) $$\phi \approx \alpha (t)=\int_{t_{k}}^{t}\omega (\tau )d\tau$$

where ω(τ) is the angular rate vector in b frame. Consider the following equation,

(9) $$\alpha (t)\times \frac{d}{dt}v(t)=\frac{1}{2}\frac{d}{dt}\left(\alpha (t)\times v(t)\right)+\frac{1}{2}\left[\alpha (t)\times \frac{d}{dt}v(t)+v(t)\times \frac{d}{dt}\alpha (t)\right]$$

where $v(t)=\int_{t_{k}}^{t}f^{b}(\tau )d\tau$. Substituting Equations (8) and (9) into Equation (7) yields

(10) $$\begin{array}{ll} u^{n} & =C\left(\int_{t_{k}}^{t_{k+1}}f^{b}dt+\int_{t_{k}}^{t_{k+1}}\phi \times f^{b}dt\right)\\ & =C\left(\int_{t_{k}}^{t_{k+1}}f^{b}dt+\int_{t_{k}}^{t_{k+1}}\alpha (t)\times f^{b}dt\right)\\ & =C(v(t_{k+1})+\int_{t_{k}}^{t_{k+1}}\left\{\frac{1}{2}\frac{d}{dt}(\alpha (t)\times v(t))+\frac{1}{2}\left[\alpha (t)\times \frac{d}{dt}v(t)+v(t)\times \frac{d}{dt}\alpha (t)\right]\right\}dt)\\ & =C\left(v(t_{k+1})+\frac{1}{2}\left[\alpha (t)\times v(t)\right]{|}_{t_{k}}^{t_{k+1}}+\frac{1}{2}\int_{t_{k}}^{t_{k+1}}\left[\alpha (t)\times f^{b}+v(t)\times \omega \right]dt\right) \end{array}$$

The second term $\frac{1}{2}\left[\alpha (t)\times v(t)\right]{|}_{t_{k}}^{t_{k+1}}$ in Equation (10) is defined as the rotation error compensation term, which represents a change in velocity due to rotational motion in the update cycle. This term leads to the low-frequency component during integration and can be compensated easily. The third integral term in Equation (10) is the sculling error compensation term, which is

(11) $$\Delta_{scull}=\frac{1}{2}\int_{t_{k}}^{t_{k+1}}\left[\alpha (t)\times f^{b}+v(t)\times \omega \right]dt$$

This term represents the integration contribution of the high-frequency component and results in rectified error during velocity update especially in the sculling motions. Thus, the key of velocity error compensation is to accurately estimate and compensate the sculling term.

Based on the analytical expression of the sculling error term, it is known that when high-frequency angular and linear vibrations occur in two or more axes simultaneously, the sculling error in velocity will be generated in the inertial sensor [10,12,17]. To improve the velocity accuracy, a series of high-rate algorithms for sculling integral compensation using angle and velocity increments as algorithm inputs have been developed [25,26]. However, they are not suitable for modern inertial sensors whose outputs are the angular rate and specific force. Some researchers designed sculling error correction algorithms based on the angular rate or linearly ramping polynomial specific force models, whose coefficients are estimated through Taylor or Picard series polynomial expansion [23,24,27]. However, it is ignored that the angular rate and specific force are changing randomly and non-linearly especially in high dynamics or complicated vibration environments. In addition, the conventional three-interval or four-interval sculling correction algorithms are usually simulated under pure sculling environments without considering vibration effect, and the results are not optimal in real vibration environments.

## 3. Sculling Error Compensation Algorithm Based on Two-Time Scale Perturbation Model

### 3.1. Performance Analysis of Specific Force under Vibrations

**Figure 1:**
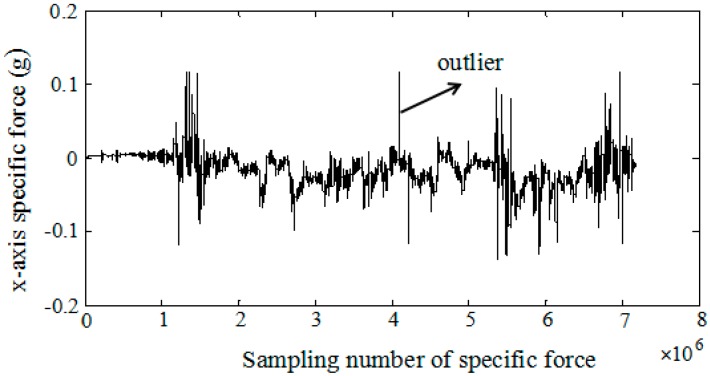
The raw specific force data of X-axis with 1.6 KHz sampling frequency.

For aircraft, engine noise and air disturbance are usually the main vibration sources [28]. The engine noise mainly depends on aircraft type. When the engine starts to work, the engine vibration is transmitted to the vehicle and affects the normal operation of onboard instruments. Air disturbance is influenced by meteorological conditions with strong random characteristics. When the aircraft flies from one irregular vortex to another in turbulent areas, the unstable air flow causes vibration. Especially, the vibration strength will be intensified if the vibration natural frequency of aircraft is equal to the vortex vibration frequency. In addition, the rotor vibration is also the main vibration source for rotor aircraft. Most vibrations can be sensed directly by the inertial components mounted on the aircraft, which lead to coning and sculling errors in attitude and velocity updates, respectively. In order to compensate the sculling error, the specific force with wide-band nonwhite noise under aircraft dynamic environments is analyzed at first. Figure 1 shows the real raw data of the X-axis accelerometer fixed on the vehicle with 1.6 KHz sampling frequency. Some outliers can be observed in the specific force measurement except noises. Thus, it is necessary to analyze the frequency spectrum of the specific force and further preprocess the specific force to improve its signal-to-noise ratio under complicated circumstances.

**Figure 2:**
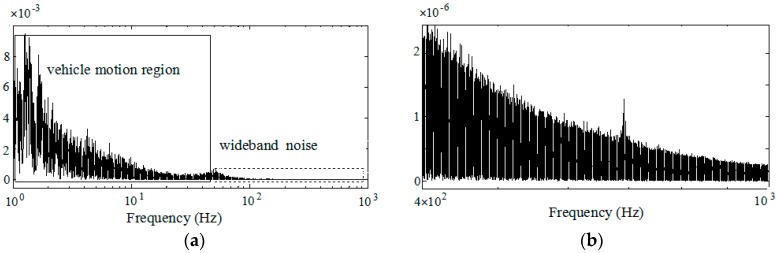
Frequency response of filtered specific force: (**a**) Frequency spectrum of filtered specific force in Figure 1; (**b**) Sensor noise region with high frequency.

Generally, a low-pass filter is often used in the preprocessing to filter high frequency noises, and enhances the signal-to-noise ratio. However, the remnant noises with high frequency still exist even after preprocessing. The result of utilizing the power spectral density (PSD) method to analyze the frequency response of filtered X-axis specific force in a random vibration experiment is shown in Figure 2a. The remnant noise with wide frequency bandwidth up to a thousand Hz is shown in Figure 2b.

It can be seen from Figure 2a that the frequency spectrum of the specific force can be partitioned into two regions under the real experimental conditions: a vehicle motion region characterized by higher dynamic energy and lower frequency bandwidth, and the wide-band noise region characterized by lower dynamic energy and higher frequency. The specific force over the sculling update interval has the characteristic with slow and fast time scale, which satisfies the requirement of the two-time scale model.

**Figure 3:**
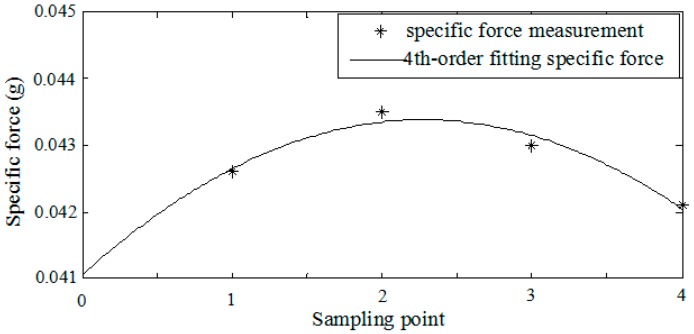
Specific force measurement and the fourth-order fitting model over one sculling update interval.

On the other hand, the Taylor series polynomial model of the specific force is usually adopted in conventional sculling algorithms to obtain the optimal coefficients of the sculling error correction term. For instance, the fourth-order fitting specific force model is often utilized in the four-interval algorithm compensating the sculling error. In fact, it is difficult for this deterministic model to describe the specific force with the slow and fast time scale characteristic under real vibration environments. Supposing the frequencies of velocity update and sculling update are 100 Hz and 400 Hz respectively, the inertial sensor’s sample frequency should be 1.6 KHz in the conventional four-interval sculling algorithm. This indicates that four sample measurements should be included in one sculling update interval. The estimates derived from the fourth-order fitting specific force model and the preprocessed specific force measurement over one sculling update interval are shown in Figure 3.

The estimates based on the fourth-order fitting specific force model cannot completely represent the real specific force characteristic over the sculling update interval. It can be seen that the errors between the estimates and real measurements can reach 0.0002 g at the second sample point and 0.0001 g at the third sample point. These errors will be accumulated after integration and cause large velocity error over time. Combing Figure 2 and Figure 3, a new model of specific force is constructed to better represent the frequency spectrum of specific force with less estimation error in Section 3.2, and the sculling error correction term based on the new model is derived in Section 3.3.

### 3.2. Two-Time Scale Perturbation Model of Specific Force

The frequency spectrum of specific force demonstrates the slow and fast characteristic time scales over the sculling update interval. In [29,30], it was found that a two-time scale model can be used to describe this experimental frequency spectrum shape. Moreover, the Markov process with weak interactions has been widely used to conveniently describe the noise in inertial sensor outputs and it can also be modeled as singularly perturbed systems [30]. Therefore, due to the simultaneous presence of slow and fast phenomena over the sculling update interval, the specific force can be characterized by a singularly perturbed process and modeled as a two-time scale perturbation model

(12) $$\varepsilon \ddot{f}(\tau )+\dot{f}(\tau )+\beta =0$$

With the boundary conditions $f(0)=f_{1}\;\mathrm{and}\;f(T)=f_{2}$, where ε is a small positive perturbation parameter; τ is the time relative to the beginning of a sculling update interval; β is a variable vector; $f_{1}$ and $f_{2}$ are the specific force vectors sensed by the accelerometer at the beginning and ending of a sculling update interval, respectively; T is the sculling update interval.

Although it is easy to determine the exact solution of Equation (12), a simplified composite solution to a two-time scale system is chosen because it can achieve nearly the same performance as the exact solution but demand less computation load [19]. From Equation (12), the singularly perturbed model of the specific force includes two subsystems: the inner subsystem and the outer subsystem. When solving the singularly perturbed model of the specific force with the boundary layer method, the simplified composite solution to Equation (12) can be obtained by solving the differential equation for each subsystem and combining their solutions.

The inner subsystem (boundary layer) is derived by stretching the time scale from τ to η=τ/ε, then $\dot{f}(\tau )=\frac{1}{\varepsilon }\dot{f}(\eta )$ and $\ddot{f}(\tau )=\frac{1}{\varepsilon^{2}}\ddot{f}(\eta )$. Substituting them into Equation (12) and letting ε=0 yield

(13) $${\ddot{f}}^{\left(i\right)}(\eta )+{\dot{f}}^{\left(i\right)}(\eta )=0\;\eta \in [0,\infty )$$

where the superscript ‘*i*’ stands for “inner”. In solving the perturbation equation, the transformed initial condition of the inner boundary layer should include the initial value of the outer subsystem, i.e., $f^{\left(i\right)}(0)=f_{1}-f^{\left(o\right)}(0)\;$, where $f^{\left(i\right)}(0)$ is the initial value of the inner subsystem, and $f^{\left(o\right)}(0)$ is the initial value of the outer subsystem. The superscript ‘*o*’ stands for “outer”. The slower vector $f^{\left(o\right)}(\tau )$ is considered as a constant equal to its initial value in the boundary layer and the faster vector $f^{\left(i\right)}(\eta )$ satisfies $\underset{\eta \to \infty }{\lim}f^{\left(i\right)}(\eta )=0$.

By solving the second-order differential Equation (13), the solution to the inner boundary layer is

(14) $$f^{\left(i\right)}(\eta )=(f_{1}-f^{\left(o\right)}(0))e^{-\eta }$$

Suppose that the parameter ε is very small. The outer subsystem of Equation (12) can be described by

(15) $${\dot{f}}^{\left(o\right)}(\tau )+\beta =0\;$$

with its terminal condition $f^{\left(o\right)}(T)=f_{2}$. Integrating Equation (15) yields the outer subsystem solution

(16) $$f^{\left(o\right)}(\tau )=-\beta \tau +\beta T+f_{2}$$

with

(17) $$f^{\left(o\right)}(0)=\beta T+f_{2}$$

The approximate composite solution of Equation (12) is constructed as the sum of the inner solution and the outer solution based on Equations (14), (16) and (17), that is

(18) $$f(\tau )=f^{\left(i\right)}(\frac{\tau }{\varepsilon })+f^{\left(o\right)}(\tau )=(f_{1}-\beta T-f_{2})e^{-\left(\frac{\tau }{\varepsilon }\right)}-\beta \tau +\beta T+f_{2}$$

The two-time scale model of angular rate can be constructed by the same method based on a singularly perturbed system. The approximate composite solution of the angular rate perturbation model over the sculling update interval can be derived as:

(19) $$\omega (\tau )=(\omega_{1}-\gamma T-\omega_{2})e^{-\left(\frac{\tau }{\varepsilon }\right)}-\gamma \tau +\gamma T+\omega_{2}$$

where γ is a vector in the angular rate singularly perturbed system; $\omega_{1}$ and $\omega_{2}$ are the angular rate vectors sensed by the gyroscopes on the vehicle at the beginning and ending time of the sculling update interval, respectively. Equations (18) and (19) are defined as the two-time scale perturbation models of inertial measurements.

In the traditional sculling compensation algorithms, two conflicting requirements usually exist. On one hand, the navigation system attempts to obtain accurate results by integrating high speed sensor data in very small step sizes, but on the other hand, this flood of data may cause great burden to the processor [31]. A good tradeoff is the key factor for sculling error compensation. It is known that the specific force model based on the polynomial in the N-interval traditional algorithm requires N new sample values over one sculling update interval, whereas the specific force based on the new two-time scale model only uses two sample values at the beginning and ending of the sculling update interval for integration. Thus, the frequency of sensor sampling based on the two-time scale model is the same as the frequency of sculling update.

### 3.3. Sculling Compensation Algorithm Based on Two-Time Scale Perturbation Models of Inertial Measurements

The two-time scale perturbation models of the specific force and angular rate are used to derive the sculling error correction term in this section. According to Equations (18) and (19), the inertial signals can be expressed in another form as follows:

(20) $$\omega (t)=ae^{-\left(\frac{t}{\varepsilon }\right)}-bt+d\;\mathrm{and}\;f(t)=Ae^{-\left(\frac{t}{\varepsilon }\right)}-Bt+D$$

where the parameters in Equation (20) are

(21) $$\begin{array}{l} a=\omega_{1}-\gamma T-\omega_{2},\;b=\gamma ,\;d=\gamma T+\omega_{2}\\ A=f_{1}-\beta T-f_{2},\;B=\beta ,\;D=\beta T+f_{2} \end{array}$$

The incremental angle vector α and incremental velocity vector v from time 0 to *t* are

(22) $$\alpha =-\varepsilon a\left(e^{-\frac{t}{\varepsilon }}-1\right)-\frac{1}{2}bt^{2}+dt,\;v=-\varepsilon A\left(e^{-\frac{t}{\varepsilon }}-1\right)-\frac{1}{2}Bt^{2}+Dt$$

Substituting Equations (21) and (22) into Equation (11), the sculling error correction term over the sculling update interval based on the proposed models is derived as

(23) $$\begin{array}{ll} {\hat{\Delta }}_{scull} & =\frac{1}{2}\int_{t_{k}}^{t_{k+1}}\left(v\times \omega +\alpha \times f\right)dt\\ & =\frac{1}{2}(A\times b-B\times a)(\frac{1}{2}\varepsilon T^{2}+2\varepsilon^{3})+\frac{1}{2}(D\times a-A\times d)(2\varepsilon^{2}-\varepsilon T)+\frac{1}{12}(B\times d-D\times b) \end{array}$$

Substituting Equation (21) into Equation (23) yields

(24) $$\begin{array}{l} A\times b-B\times a=(f_{1}-f_{2})\times \gamma +(\omega_{1}-\omega_{2})\times \beta \\ D\times a-A\times d=f_{2}\times \omega_{1}-f_{1}\times \omega_{2}+\gamma T\times f_{1}+\beta T\times \omega_{1}\\ B\times d-D\times b=\beta \times \omega_{2}-f_{2}\times \gamma \end{array}$$

A numerical algorithm for computing the sculling integral term is developed based on Equations (23) and (24). In the conventional four-interval algorithm based on the fourth-order fitting, the sculling compensation term is [32]

(25) $$\begin{array}{ll} {\hat{\Delta }}_{scull}= & K_{v1}\left[\Delta \theta_{1}\times \Delta v_{2}+\Delta \theta_{3}\times \Delta v_{4}+\Delta v_{1}\times \Delta \theta_{2}+\Delta v_{3}\times \Delta \theta_{4}\right]\\ & +K_{v2}\left[\Delta \theta_{1}\times \Delta v_{3}+\Delta \theta_{2}\times \Delta v_{4}+\Delta v_{1}\times \Delta \theta_{3}+\Delta v_{2}\times \Delta \theta_{4}\right]\\ & +K_{v3}\left[\Delta \theta_{1}\times \Delta v_{4}+\Delta v_{1}\times \Delta \theta_{4}\right]+K_{v4}\left[\Delta \theta_{2}\times \Delta v_{3}+\Delta v_{2}\times \Delta \theta_{3}\right] \end{array}$$

where $K_{v1}=\frac{736}{945}$, $K_{v2}=\frac{334}{945}$, $K_{v3}=\frac{526}{945}$, and $K_{v4}=\frac{654}{945}$ are the coefficients derived from the fourth-order fitting model of inertial information. In order to calculate the sculling compensation term in the conventional four-interval algorithm, four new inertial measurements about angular and linear motions should be supplied in one sculling interval $\Delta \theta_{1},\;\Delta \theta_{2},\;\Delta \theta_{3},\;\Delta \theta_{4}$ are angular increments and $\Delta v_{1},\;\Delta v_{2},\;\Delta v_{3},\;\Delta v_{4}$ are velocity increments at the four different sampling times during one sculling interval. It is convenient to obtain the sculling compensation term according to Equation (25) utilizing the inertial sensors whose outputs are the angular increment and velocity increment. However, the measurements of most inertial sensors nowadays are the angular rate and specific force. The angles and velocities at the sampling times can be calculated by integrating the measurements of the angular rate and specific force, and then the angular and velocity increments can be obtained by further processing of the calculated angles and velocities, which will introduce more calculation errors.

By comparing Equations (23) and (25), it can be seen that the conventional sculling algorithm utilizes angular and velocity increments to compute the sculling correction term, while the proposed sculling algorithm directly uses the angular rate and specific force as inputs and estimates the sculling correction term based on their two-time scale perturbation models. Therefore, the proposed approach is more appropriate for the modern accelerometer with the specific force output and gyroscope with the angular rate output. Additionally, the proposed approach can complete the sculling compensation with less sample measurements compared with the conventional four-interval algorithm that requires more sensor sampling data. In particular, in one sculling update interval, the proposal algorithm needs two sample measurements from each sensor ($\omega_{1}$ and $\omega_{2}$ from gyroscope, and $f_{1}$ and $f_{2}$ from the accelerometer), whereas the conventional four-interval algorithm needs four sample measurements ($\Delta \theta_{1},\;\Delta \theta_{2},\;\Delta \theta_{3},\;\Delta \theta_{4}$ for angular measurements and $\Delta v_{1},\;\Delta v_{2},\;\Delta v_{3},\;\Delta v_{4}$ for velocity measurements). In addition, tuning the parameter ε and vectors β and γ can effectively improve the accuracy of the proposed sculling correction algorithm. The parameter selection rules will be given in the next section.

### 3.4. Selection of Perturbation Model Parameters Based on Gravitational Search Optimization Method

The parameter ε>0 is a sufficiently small constant that represents the separation of time scales between the fast dynamics and the slow dynamics of inertial sensor outputs [19]. The vector parameters β and γ describe the vehicle specific force and angular rate motion in the sculling update interval.

Among many optimization algorithms, the gravitational search algorithm based on Newton’s law of universal gravitation can perform the global search in the solution space [33]. In this algorithm, each particle is regarded as a mass object in the solution space and it moves in the gravitational field of all other particles. The particles with small or large fitness will approach the optimal solution at a slow or fast speed, respectively. In order to determine the parameters ε, β, and γ, the gravitational search optimization algorithm is used due to its excellent search speed and ability to tune and obtain the optimal solution.

**Figure 4:**
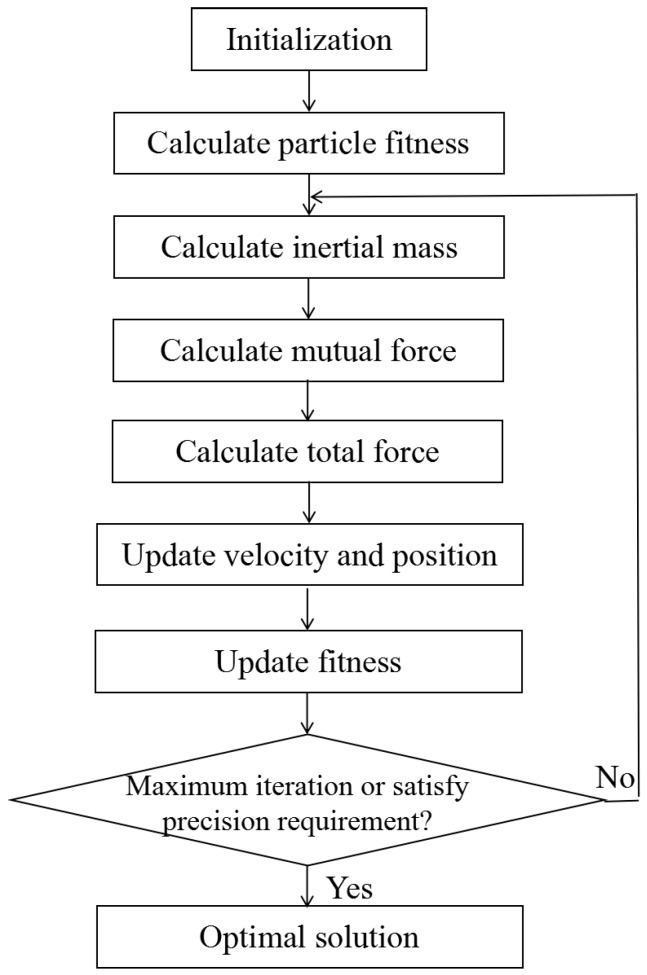
Gravitational search optimization algorithm.

The parameters ε, β and γ in the two-time scale perturbation models of inertial measurements are considered as mass particles and optimized by the gravitational search algorithm. Define the fitness function as follows

(26) $$F_{fit}=\frac{1}{N}\sum_{i=1}^{N}\left[{\left(X_{i}-{X_{i}}^{\prime }\right)}^{2}+{\left(Y_{i}-{Y_{i}}^{\prime }\right)}^{2}\right]$$

where *N* is the number of mass particles. $X_{i}$ and $Y_{i}$ are inertial measurements of the specific force or angular rate corresponding to the *i*th particle. ${X_{i}}^{\prime }$ and ${Y_{i}}^{\prime }$ are their estimates based on the two-time scale perturbation models. The flowchart of the parameter optimization algorithm is shown in Figure 4.

First, initialize the velocity and position of mass particles in the three dimensions randomly. Calculate the fitness values of all mass particles by Equation (26). According to Equation (27), the inertial mass of each mass particle is updated using the fitness value when the particle is moving:

(27) $$m_{i}\left(t\right)=\frac{fit_{i}\left(t\right)-worst\left(t\right)}{best\left(t\right)-worst\left(t\right)},\;M_{i}\left(t\right)=m_{i}\left(t\right)/\sum_{j=1}^{N}m_{j}\left(t\right)$$

where $M_{i}\left(t\right)$ denotes the inertial mass of each particle at the *t*th iteration. $fit_{i}\left(t\right)$ is the fitness value of the *i*th particle at the *t*th iteration. best(t) and worst(t) are the minimum and maximum fitness values of all particles at the *t*th iteration, respectively.

Next, calculate the mutual force acting on the mass particle with the inertial mass based on Newton’s law of universal gravitation. The force of the *j*th particle on the *i*th particle is

(28) $$F_{ij}=G\left(t\right)\frac{M_{j}\left(t\right)M_{i}\left(t\right)}{R_{ij}\left(t\right)}\left(x_{j}\left(t\right)-x_{i}\left(t\right)\right)\;,\;G\left(t\right)=G_{0}e^{\left(-\lambda /\xi \right)}$$

where G(t) denotes the gravitational coefficient changing with current iteration number λ. $G_{0}$ is the initial value of the gravitational coefficient and ξ is the maximum iteration number. $M_{i}\left(t\right)$ and $M_{j}\left(t\right)$ represent the inertial mass of the *i*th particle and the *j*th particle respectively at the *t*th iteration. $x_{i}\left(t\right)$ and $x_{j}\left(t\right)$ are the positions of $M_{i}\left(t\right)$ and $M_{j}\left(t\right)$ in the space at the *t*th iteration, and they also represent the values of the perturbation parameters at the *t*th iteration. $R_{ij}\left(t\right)$ is the Euclidean distance between $M_{i}\left(t\right)$ and $M_{j}\left(t\right)$. Therefore, the total force on the *i*th particle in the three-dimensional space is

(29) $$F_{i}\left(t\right)=\sum_{j=1,j\neq i}^{N}F_{ij}\left(t\right)$$

Then update the particle velocity $v_{i}\left(t+1\right)$ and position $x_{i}\left(t+1\right)$ based on the total force on the *i*th particle according to Equation (30):

(30) $$v_{i}\left(t+1\right)=v_{i}\left(t\right)+\frac{F_{i}\left(t\right)}{M_{i}\left(t\right)},x_{i}\left(t+1\right)=x_{i}\left(t\right)+v_{i}\left(t+1\right)$$

Finally, the new fitness value is updated and used as the condition. The optimal parameter ε, β and γ are obtained if the current iteration number reaches the preset maximum iteration number, or the precision of the updated fitness satisfies the preset requirement that makes the mutual force between mass particles the largest. If the condition is not met, the algorithm moves to the next iteration.

Due to the wide bandwidth of sensor measurement noise, the resulting optimal parameter ε is 0.00001 after the search. A series of γ and β are obtained as well and the fitting formula of γ and β are set to be

(31) $$\gamma =(\omega_{2}-\omega_{1})\times 0.001\;\mathrm{and}\;\beta =(f_{2}-f_{1})\times 0.001$$

The formulas are based on the driving experimental data of the fiber IMU with 400 Hz sample frequency. However, they can change for different vehicles working in different environments.

## 4. Simulation under Wideband Vibrations and Analysis

Simulations were performed to illustrate the advantages of the proposed sculling algorithm compared with the conventional four-interval sculling algorithm under the complex vibration environment. In the simulations, the vehicle was assumed to undergo the sculling motion that is considered the worst dynamic environment for SINS since the sculling error is maximized under this motion. In traditional algorithms, the simulation condition of pure sculling motion does not take into account random noises. However, the advantage of the proposed algorithm is that it can sufficiently correct both the sculling and pseudo sculling velocity errors usually caused by vibration noise. The simulation focuses on the sculling error compensation affected by vibrations of different frequencies. According to the analysis of experimental data given in Figure 2a, harmonic angular and linear vibrations with the same frequency are constructed as the simulation condition. The vibrations with noises are acting along the vehicle orthogonal axes:

(32) $$\omega (t)=\left[\begin{array}{c} A_{\alpha }\Omega \cos(\Omega t)+A_{\alpha n}\Omega_{n}\cos(\Omega_{n}t)\\ 0\\ 0 \end{array}\right],\;f(t)=\left[\begin{array}{c} 0\\ A_{p}\sin(\Omega t)+A_{pn}\sin(\Omega_{n}t)\\ 0 \end{array}\right]$$

where $A_{\alpha }$ and $A_{p}$ denote the amplitude of the angular and linear vibrations, respectively. Ω is the angular and linear vibration frequency. $A_{\alpha n}$ and $A_{pn}$ represent the amplitude of noise existing in the angular and linear vibrations, respectively. $\Omega_{n}$ is the noise frequency.

The angular and velocity increment over one sculling update interval can be determined by integrating Equation (32),

(33) $$\begin{array}{l} \alpha (t)=\int_{t_{k}}^{t_{k+1}}\omega dt=\left[\begin{array}{c} A_{\alpha }\left(\sin\Omega t_{k+1}-\sin\Omega t_{k}\right)+A_{\alpha n}\left(\sin\Omega_{n}t_{k+1}-\sin\Omega_{n}t_{k}\right)\\ 0\\ 0 \end{array}\right]\\ v(t)=\int_{t_{k}}^{t_{k+1}}fdt=\left[\begin{array}{c} 0\\ \frac{A_{p}}{\Omega }\left(\cos\Omega t_{k}-\cos\Omega t_{k+1}\right)+\frac{A_{pn}}{\Omega }\left(\cos\Omega_{n}t_{k}-\cos\Omega_{n}t_{k+1}\right)\\ 0 \end{array}\right] \end{array}$$

**Figure 5:**
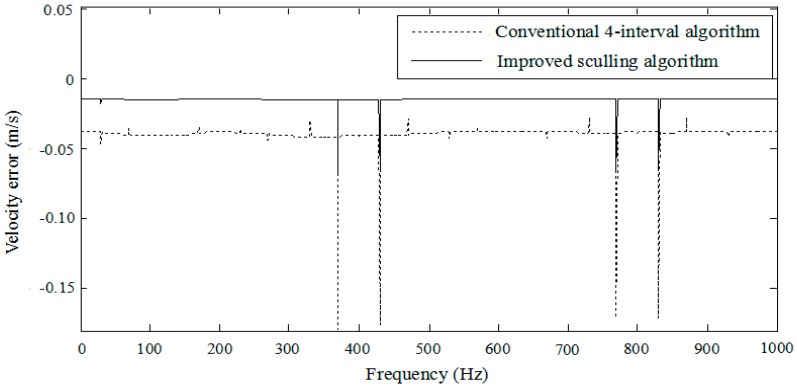
Velocity error.

**Figure 6:**
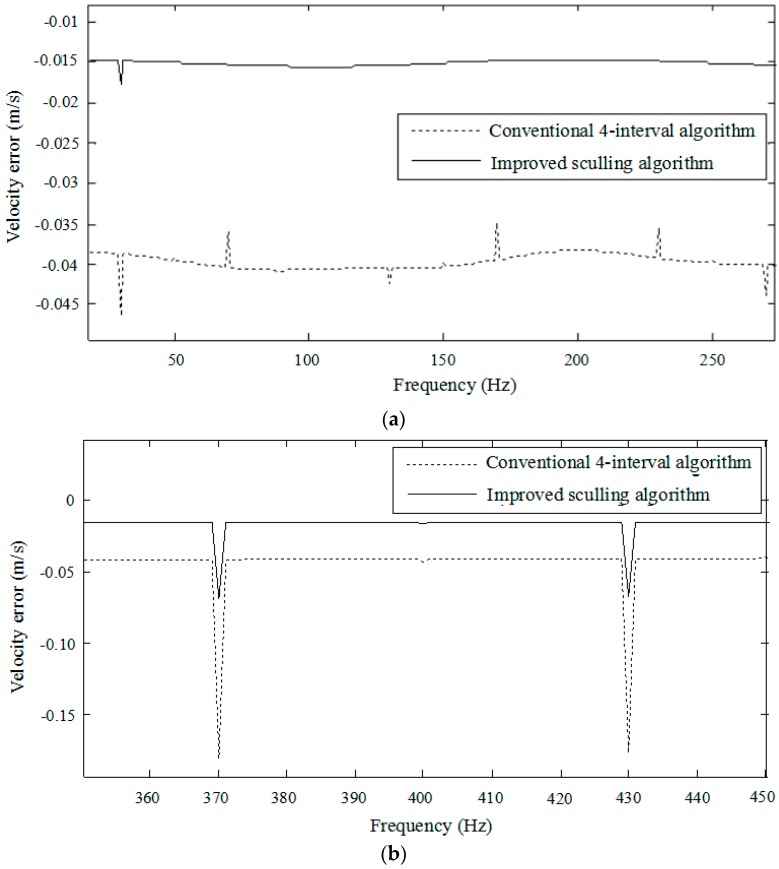
Velocity errors at the low frequency and high frequency: (**a**) Velocity error at the low frequency; (**b**) Velocity error at the high frequency.

Substituting Equation (33) into the sculling term leads to the theoretic sculling compensation term $\Delta_{scull}$. In simulation, the angular and linear vibration amplitudes of the vehicle sculling motion are assumed $A_{\alpha }=0.1^{\circ }$ and $A_{p}=0.0001\;g$, respectively. The angular and linear vibration frequency Ω is 30 Hz. The noise frequency $\Omega_{n}$ varies in a wide range from 0 Hz to 1000 Hz. The angular noise amplitude is $A_{\alpha n}=0.01^{\circ }$ and the linear velocity noise amplitude is $A_{pn}=0.00001\;g$. The parameters γ and β are determined according to Equation (31). The outputs of strapdown inertial sensors are the angular rate and specific force with the sample frequency 400 Hz. The total duration of simulation is 10 s. The results of the velocity error along the Z-axis in the navigation frame are illustrated in Figure 5 and Figure 6. Note that the sculling update frequency of the improved sculling algorithm is 400 Hz as well, while that of the conventional four-interval algorithm is 100 Hz when the sensor sample frequency is 400 Hz.

Figure 5 reveals that there is a pseudo sculling phenomenon caused by the overlap of frequency bands. It can be seen that the velocity error is actually amplified around 400 Hz and 800 Hz when the multiples of the sample frequency are mixed with the noise frequency. It is evident that the improved algorithm performs much better for the velocity update than the conventional four-interval approach under the sculling motion, because the improved algorithm can reduce the pseudo sculling error.

Figure 6 is presented to more clearly compare the performances of both algorithms at around the low noise frequency from 0 Hz to 250 Hz and the high noise frequency from 350 Hz to 450 Hz. It can be seen that the average velocity error of the improved sculling algorithm is 0.015 m/s at the low frequency band while the average velocity error of the conventional four-interval algorithm reaches 0.04 m/s. The maximum velocity error of the conventional four-interval algorithm in the vicinity of 400 Hz is 0.1795 m/s while that of the improved algorithm reduces to 0.0685 m/s under the same simulated condition. The improved sculling algorithm is more effective in attenuating the pseudo sculling error than the conventional algorithm as the frequency increases.

If the error drift of an algorithm is minimized in the sculling motion, it will perform satisfactorily in most other environments. In the process of sculling error compensation, the error drift is used as the criterion for accuracy evaluation, and its definition is given in Equation (34) [34],

(34) $$\rho_{drift}={\hat{\Delta }}_{scull}-\Delta_{scull}$$

**Figure 7:**
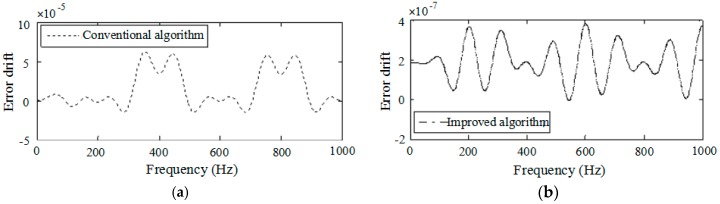
Error drifts: (**a**) Error drift of the conventional algorithm; (**b**) Error drift of the improved algorithm.

The results of error drift simulations under the sculling motion are shown in Figure 7.

Figure 7 illustrates that the error drifts of the conventional and improved algorithms change with the sculling motion noise frequency. The error drift of the improved algorithm based on the two-time scale perturbation model of inertial measurement is much smaller than the conventional one. Especially, when the noise frequency and sensor sample frequency fall into the overlap bands around 400 Hz and 800 Hz, the improved sculling algorithm can effectively attenuate the pseudo sculling error arising from the inertial sensor errors and vibration.

The average velocity error during the simulation duration is used to evaluate the accuracy performance of the two algorithms. The computational time is the total time that the algorithms spent on calculating the sculling error term in one velocity update interval. The performance statistic result is shown in Table 1.

It is evident that the improved algorithm is more accurate than the four-interval algorithm. Besides favorable accuracy, the improved algorithm shows its excellent work efficiency compared with the conventional algorithm at the same sample frequency. It can be seen that the computational time of the improved algorithm is much less than the conventional one. Furthermore, when the vehicle has very fast motion, especially a vibration-like oscillating motion, the sculling error can creep into velocity if the integration is not fast enough. Then it is necessary to design a fast integration algorithm to compensate the sculling error. If fast integration is desired to avoid the velocity sculling error, inertial data at a very high sample rate should be applied, yet it will impose a heavy burden on the processor. From Table 1, it can be seen that the update frequency of the new sculling compensation based on the two-time scale perturbation model is equal to the inertial sensor sample frequency 400 Hz, and the accuracy of the improved algorithm is higher. The update frequency of the conventional four-interval algorithm is 100 Hz because four sample data is needed to calculate the polynomial model coefficients in one sculling update interval. The statistical results indicate that the improved sculling correction algorithm based on the two-time scale perturbation model can effectively reduce the sculling error with less computation load.

## 5. Conclusions

In this paper, a novel velocity update algorithm for sculling error compensation was proposed for SINS based on the inertial sensor outputs of the angular rate and specific force. The new algorithm utilizes the inertial information’s two-time scale singular perturbation models instead of polynomial models to compute the increments of the velocity and angle to compensate the sculling error in the velocity update. Experimental results have shown that the new sculling compensation algorithm based on the singular perturbation can reduce the algorithm design complexity and achieve more accurate performance with less computation load compared with the traditional algorithm. In addition, the proposed algorithm can decrease both the sculling and pseudo sculling velocity errors that are usually caused by stochastic vibrations. Owing to these advantages, the improved algorithm is more suitable for SINS.

## Figures and Tables

**Table 1:** Performance comparison.

Performance Index	Four-Interval Algorithm	Improved Algorithm
Accuracy (m/s)	−0.0399	−0.0152
Computational time (s)	0.01432	0.00414
Correction frequency (Hz)	100	400
